# Single-Pass Tangential Flow Filtration (SPTFF) of Nanoparticles: Achieving Sustainable Operation with Dilute Colloidal Suspensions for Gene Therapy Applications

**DOI:** 10.3390/membranes13040433

**Published:** 2023-04-15

**Authors:** Akshay S. Chaubal, Andrew L. Zydney

**Affiliations:** Department of Chemical Engineering, Pennsylvania State University; University Park, PA 16802, USA

**Keywords:** SPTFF, ultrafiltration, continuous processing, viral vectors, lentivirus, nanoparticles, gene therapy, critical flux, concentration polarization

## Abstract

Recent approval of several viral-vector-based therapeutics has led to renewed interest in the development of more efficient bioprocessing strategies for gene therapy products. Single-Pass Tangential Flow Filtration (SPTFF) can potentially provide inline concentration and final formulation of viral vectors with enhanced product quality. In this study, SPTFF performance was evaluated using a suspension of 100 nm nanoparticles that mimics a typical lentivirus system. Data were obtained with flat-sheet cassettes having 300 kDa nominal molecular weight cutoff, either in full recirculation or single-pass mode. Flux-stepping experiments identified two critical fluxes, one based on boundary-layer particle accumulation (J_bl_) and one based on membrane fouling (J_foul_). The critical fluxes were well-described using a modified concentration polarization model that captures the observed dependence on feed flow rate and feed concentration. Long-duration filtration experiments were conducted under stable SPTFF conditions, with the results suggesting that sustainable performance could potentially be achieved for as much as 6 weeks of continuous operation. These results provide important insights into the potential application of SPTFF for the concentration of viral vectors in the downstream processing of gene therapy agents.

## 1. Introduction

The recent success of nucleic-acid-based biotherapeutics has led to a resurgence of interest in gene therapy to provide treatments for many debilitating diseases [1]. Currently, there are more than 20 cell and gene therapy products that have been approved by the FDA for treatments ranging from hemophilia to cancer [2]. Viral vectors serve as efficient delivery vehicles for targeted gene therapy applications since they can effectively transfect a broad range of tissues [3]. Lentiviruses (LVs) are of particular interest given their ability to provide long-term gene expression in both dividing and non-dividing cells [4]. The FDA recently approved betibeglogene autotemcel (for treatment of severe β-thalassemia) and elivaldogene autotemcel (for treatment of cerebral adrenoleukodystrophy), both of which use lentiviral vectors to permanently modify the patient’s hematopoietic stem cells [5,6].

One of the major challenges in the commercialization of these innovative therapies is the need for significant concentration of the dilute lentivirus as part of the downstream process [7]. Zimmerman et al. [8] demonstrated that ultrafiltration (UF) could provide more than 10-fold concentration of lentiviral vectors, although the lentivirus recovery for the two-step process involving a membrane adsorber and UF was less than 50%. Papanikolau et al. [9] were able to obtain lentivirus titers of 7 × 10^8^ infectious particles/mL using small-scale Amicon Ultra centrifugal ultrafilters. Several studies have demonstrated the use of tangential flow filtration (TFF) to concentrate lentivirus in larger-scale flat-sheet cassettes [7,10,11,12]. Concentration factors as high as 30–40× and particle recoveries around 90% have been reported during TFF with LVs [11], although the percent recovery was evaluated based on the presence of p24 capsid protein, which does not account for any loss of infectivity that might have occurred due to repeated passage of the shear-sensitive lentivirus [13] through the TFF module and pumps [14,15].

An attractive alternative to conventional TFF is to use Single-Pass Tangential Flow Filtration (SPTFF), in which the product is concentrated along the length of the module in a single pass without any recycling [16]. In addition to providing potential improvements in product quality, SPTFF can lead to reductions in manufacturing costs and greater manufacturing flexibility [17]. SPTFF has been used for inline concentration of monoclonal antibodies (mAbs), both to improve the productivity of chromatographic separations [18,19] and to reduce process volumes to minimize tankage constraints [20,21]. In addition, Yehl and Zydney demonstrated that SPTFF could be effectively used for continuous concentration of a model mAb for 126 h, without any membrane regeneration, by operating hollow fiber modules at a sufficiently low filtrate flux [22]. Although the presence of a “critical flux” during TFF, below which there is negligible fouling, is well-established [23,24,25,26], there have been no previous studies of the flux or fouling behavior of lentivirus particles. Arunkumar and Singh examined the ultrafiltration behavior of an adeno-associated virus (AAV), which was about 30 nm in diameter compared to the 100 nm LV, and identified a critical TMP where the resistance caused by particle accumulation within the concentration polarization boundary layer becomes significant [27]. Wolf et al. used a particle-friction-based model to calculate the critical flux during tangential flow filtration of HIV-virus-like particles (155 nm in size) [28], although this model tended to significantly underpredict the critical flux. Both studies highlighted the importance of operating below a critical flux for sustainable operation with dilute colloidal suspensions, but the data were focused on traditional TFF operation.

This study explores the use of SPTFF for the filtration of lentiviral vectors using spherical 100 nm nanoparticles as a model system based on the reported size and spherical shape of lentivirus [13]. Several previous studies have used nanoparticles as viral surrogates in membrane filtration. This includes the removal of virus-sized particles from water [29,30,31], as well as the use of polystyrene latex nanoparticles as models for rhabdovirus [32], live-attenuated virus [33], and adeno-associated viral vectors [34]. In addition to their use as a model virus, nanoparticles have also been explored as vehicles for targeted drug delivery [35], with ultrafiltration used to concentrate the product and standardize formulation properties [36,37,38,39]. Ultrafiltration has been studied for the concentration of polyethylene oxide-stabilized nanoparticle delivery systems [40], and as a method of separating free drug from lipid and polymeric nanocarriers [41].

Flux-stepping experiments were used to identify two distinct values for the critical flux: one associated with particle loss due to accumulation in the concentration polarization boundary layer, and one associated with a pressure increase due to membrane fouling. The critical flux data were effectively described using a concentration polarization model, with the calculated value of the critical wall concentration corresponding to a particle volume fraction of ≈5%. Operating the SPTFF module below the critical flux enabled continuous operation for 24 h with less than 0.1 psi increase in TMP, suggesting that SPTFF could be successfully employed for continuous concentration of lentiviral vectors for extended periods of time without any need for membrane regeneration or replacement.

## 2. Materials and Methods

### 2.1. Nanoparticles

Experimental studies were performed using FluoSpheres^®^ 100 nm fluorescent carboxylate-modified polystyrene latex microspheres (Thermo Fisher Scientific, Waltham, United States) as a model lentivirus system. The stock nanoparticles were suspended in distilled water and provided at a concentration of 2% solids. Nanoparticle feed suspensions were prepared by diluting the stock suspension in 10 mM phosphate-buffered saline (PBS) at pH 7.4 (Thermo Fisher Scientific, Waltham, United States). Prior to use, feed suspensions were sonicated in an ultrasonic cleaner bath (VWR International, Radnor, United States) at an operating frequency of 35 kHz for 15 min to break apart any aggregates that might have formed during storage/preparation.

Nanoparticle concentrations were determined from their fluorescence intensity, evaluated using a Tecan Infinite m200 Pro microplate reader (Tecan, Männedorf, Switzerland) at excitation and emission wavelengths of 580 and 605 nm, respectively. The size distribution and zeta potential were evaluated by both dynamic light scattering (DLS) using a Zetasizer Nano ZS (Malvern Panalytical, Malvern, United Kingdom), and nanoparticle tracking analysis (NTA) using a ZetaView Particle Tracking Analyzer (ParticleMetrix GmbH, Munich, Germany). Zeta potential measurements were performed using both PBS at pH 7.4 and a 50 mM tris, 5% sucrose, and 50 mM L-arginine buffer at pH 7.5, since those conditions have been used previously for lentivirus characterization [42].

### 2.2. Experimental Setup

All experiments were performed using Pellicon^®^ 3 cassettes with Ultracel^®^ regenerated cellulose membranes having a nominal molecular weight cutoff of 300 kDa and total membrane area of 88 cm^2^ (MilliporeSigma, Burlington, United States). The module contained two membranes, separated by a C-screen.

Cassettes were mounted in a Pellicon^®^ Mini Cassette Holder (MilliporeSigma, Burlington, United States) and torqued to 190 inch-pounds to prevent leakage. The membrane module was connected to two Masterflex^®^ L/S^®^ peristaltic pumps (Avantor, Radnor, United States) to control the feed and permeate flow rates. The transmembrane pressure (TMP) was measured using Ashcroft^®^ digital pressure gauges connected to the feed inlet and permeate outlet ports (the retentate outlet was open to the atmosphere):

(1) $$\mathrm{TMP}=\frac{P_{f}+P_{r}}{2}-P_{p}$$

with P_f_, P_r_, and P_p_ corresponding to the pressures at the feed inlet, retentate outlet, and permeate outlet, respectively.

**Figure 1:**
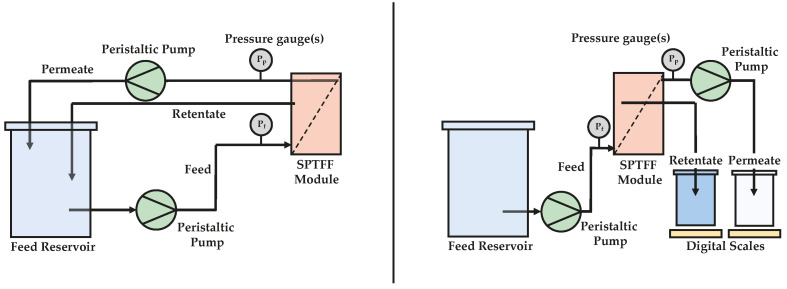
Full recirculation (**left**) and single-pass (**right**) experimental setup.

SPTFF experiments were conducted in both full recirculation and single-pass mode. In full recirculation mode, both the retentate and permeate outlet streams were recycled back to the feed reservoir, whereas in single-pass mode, the outlet streams were sent to their own separate collection vessels (as illustrated in Figure 1).

## 3. Results and Discussion

### 3.1. Nanoparticle Characterization

The average hydrodynamic diameter of the polystyrene latex carboxylate-modified nanoparticles was 122 nm measured by Dynamic Light Scattering (DLS) and 104 nm measured by Nanoparticle Tracking Analysis (NTA). The larger size in DLS is due to weighting by the intensity, which scales strongly with particle size. The zeta potential in 50 mM tris, 5% sucrose, and 50 mM L-arginine at pH 7.5 was –25 mV, consistent with the carboxylation of the nanoparticle surface. These values are similar to those reported in the literature for a lentiviral vector under comparable conditions as shown in Table 1 [42]. It was not possible to evaluate the zeta potential in the 10 mM PBS, but the zeta potential in 1 mM PBS was considerably more negative due to the lower ionic strength compared to that in the tris buffer. 

### 3.2. Particle Loss during SPTFF

**Figure 2:**
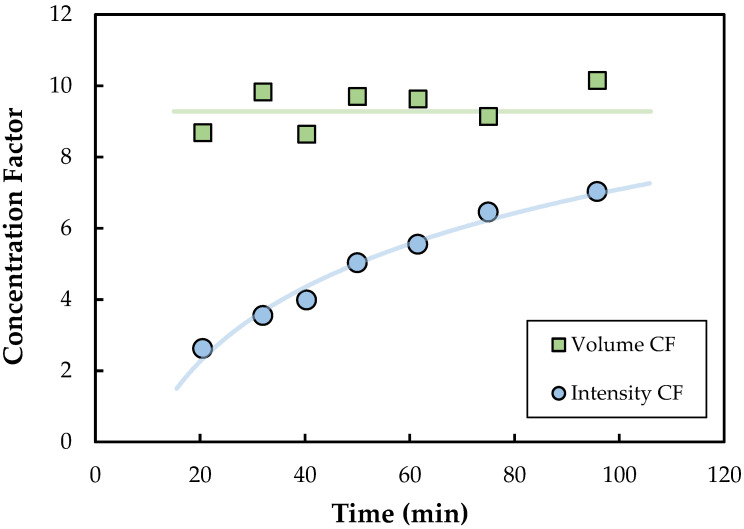
Concentration factors determined from the measured flow rates (volume CF) and fluorescence intensities (intensity CF) during an SPTFF experiment performed with the Pellicon^®^ 3 module using a feed with 3 × 10^10^ particles/mL at a feed flux of 33.1 LMH and a permeate flux of 29.5 LMH.

Typical data for an SPTFF experiment (run in single-pass mode) for a feed suspension containing 3 × 10^10^ particles/mL performed at a feed flux of 33.1 L/m^2^/h (LMH) and a permeate flux of 29.5 LMH, corresponding to a conversion of 89%, are shown in Figure 2. The 300 kDa membranes used in this study were fully retentive to the nanoparticles, with no nanoparticles detected in the permeate solution under any conditions. The concentration factor was evaluated from both the measured particle concentrations in the feed and exit retentate streams (determined by fluorescence intensity) and the measured feed and retentate flow rates (determined by timed collection). The volume concentration factor (volume CF), which was determined from the ratio of the inlet to exit flow rates, remained constant throughout the experiment, with an average value of 9.2. In contrast, the intensity concentration factor (intensity CF), which was determined from the ratio of fluorescence intensity in the retentate exit to that in the feed, was well below that determined from the flow rates, and increased from <3 at t = 20 min to ≈7 after 100 min. This is a direct result of the accumulation of nanoparticles within the SPTFF module, with the rate of particle accumulation decreasing with time, i.e., as capture sites on the membrane and within the module become saturated. This particle accumulation was easily visible in modules that were cut open after the filtration experiment, with bright fluorescence observed over the entire length of the module (Figure S1).

To determine the nature of the particle accumulation, a series of experiments were performed in which the permeate line was periodically clamped to eliminate the permeate flux (which drives particles towards the membrane surface). In each case, clamping the permeate caused a significant release of particles from the module (detected by a spike in fluorescence intensity in samples obtained from the retentate exit), suggesting that particle accumulation occurs primarily on the membrane surface (as opposed to within the spacer), with the deposited particles only loosely adherent to the membrane.

### 3.3. Critical Flux Behavior

Additional insights into the origin of the particle loss seen in Figure 2 were obtained using a modified flux-stepping procedure. Experiments were performed with nanoparticle feed suspensions at concentrations between 2 and 4 × 10^10^ particles/mL with the modules operated in full recirculation mode, i.e., with both the retentate and the permeate exit lines recycled to the feed reservoir. The feed flow rate was maintained at a constant value, while the permeate flux was increased stepwise every 40–60 min by adjusting the pump on the permeate exit line.

**Figure 3:**
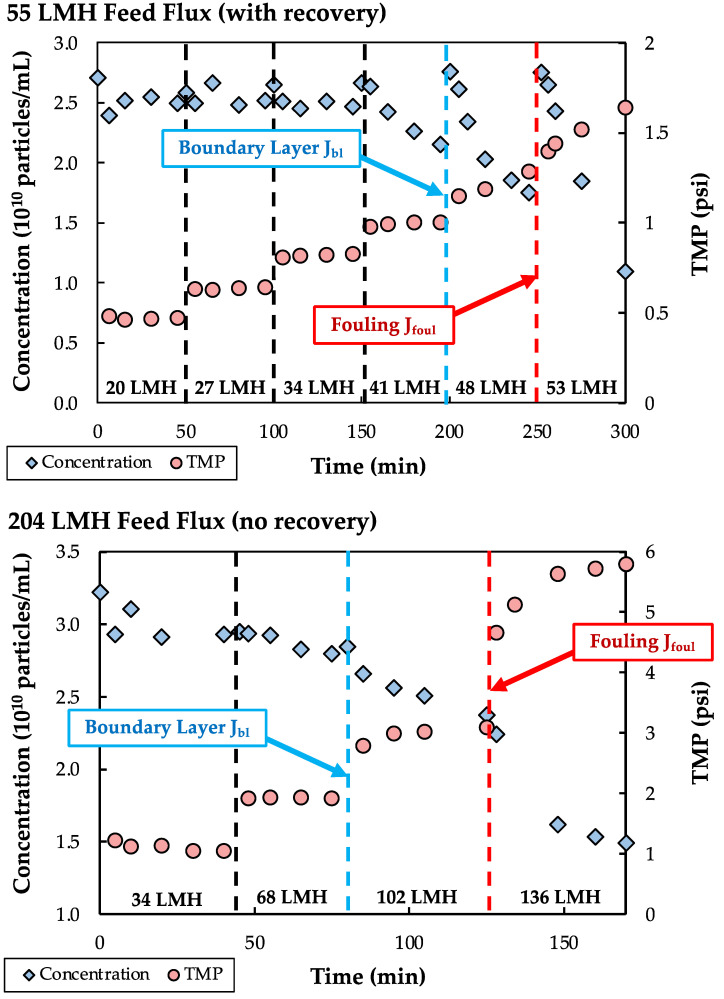
Flux-stepping experiments at feed fluxes of 55 (**top** panel) and 204 LMH (**bottom** panel) using single Pellicon^®^ 3 modules with a feed concentration between 2.5 and 3.0 × 10^10^ particles/mL.

Typical results for the TMP and particle concentration in the feed reservoir during two separate flux-stepping experiments are shown in Figure 3 at feed flow rates of 8 and 30 mL/min, corresponding to feed fluxes of 55 and 204 LMH. The experiment conducted at 55 LMH included a recovery period in which the feed was circulated through the module for approximately 5 min (with the permeate port closed) between each flux step to resuspend any nanoparticles that were weakly adhered to the membrane. This recovery step was not required for the run at 204 LMH, since the higher shear rate kept the nanoparticles well suspended. At low permeate fluxes, both the feed concentration and the TMP remained constant in each interval. However, when the permeate flux was increased beyond a critical value, there was a noticeable decline in the feed concentration due to particle accumulation within the membrane module. This particle accumulation did not, at least immediately, give rise to an increase in TMP. Instead, the TMP began to show a significant increase with time at somewhat higher values of the permeate flux. This unusual behavior is likely a result of the very dilute nanoparticle suspensions used in these experiments; the 3 × 10^10^ particles/mL feed corresponds to a particle volume fraction of <0.00003. Our hypothesis is that the initial reduction in particle concentration is due to the accumulation of particles in a concentration polarization boundary layer that forms adjacent to the membrane, but this boundary layer only causes a significant increase in TMP when there is sufficient buildup of particles to provide a substantial additional resistance to transmembrane flow.

Based on these observations, we defined two different values of the critical flux during SPTFF of these dilute nanoparticle suspensions: the boundary-layer critical flux (J_bl_) was defined as the flux at which greater than 25% particle loss was observed, while the critical flux for fouling (J_foul_) was defined as the flux at which the TMP gradient exceeds 0.005 psi/min (corresponding to a 7 psi increase over 24 h of operation). In each case, the critical fluxes were evaluated as the average of the flux values during operation immediately above and below the transition. Note that a similar approach to determining the critical flux was used by Kwon et al. [25] for the filtration of much larger particles (0.46 to 11.9 µm), with J_bl_ described as the “mass balance” critical flux based on a change in particle concentration in the feed reservoir.

**Figure 4:**
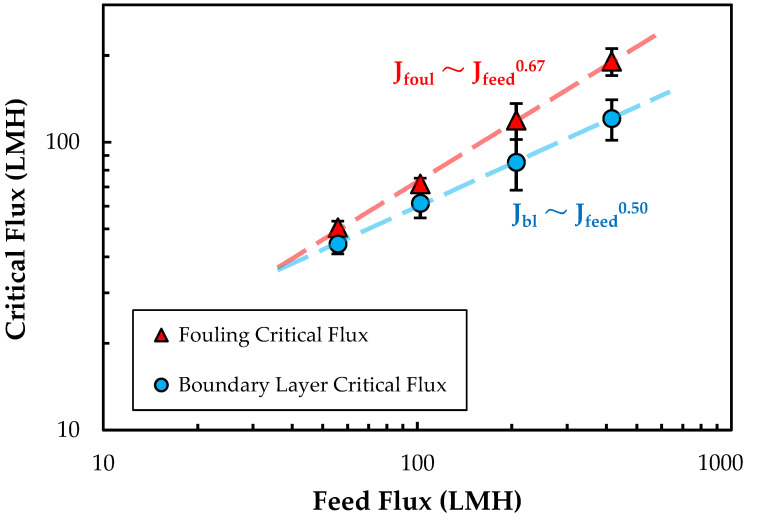
Boundary-layer (J_bl_) and fouling (J_foul_) critical fluxes as a function of feed flux (J_feed_) for single Pellicon^®^ 3 modules with a feed concentration of ≈2.5 × 10^10^ particles/mL. Error bars represent the range in flux values just above and below the transition point.

Figure 4 shows the measured values of the boundary-layer and fouling critical fluxes as a function of feed flux. Both critical fluxes increase with increasing feed flux (J_feed_), which is likely due to the reduction in the extent of particle polarization associated with the higher shear rates within the module. Both J_bl_ and J_foul_ show a power-law dependence on the feed flux (linear relationship on the log-log plot), with exponents of 0.50 ± 0.01 and 0.67 ± 0.02, respectively. The stronger dependence of the fouling critical flux on the feed flux means that the difference between J_bl_ and J_foul_ increases with increasing J_feed_. At very low feed flow rates, it is likely that the boundary-layer accumulation occurs rapidly when the permeate flux exceeds J_bl_, leading to an almost immediate increase in TMP. This is discussed in more detail below.

### 3.4. Concentration Polarization Model

The data in the previous section suggest that the critical flux is determined by nanoparticle concentration polarization. However, classical polarization models, which predict a logarithmic dependence on the bulk particle concentration, neglect the effect of the filtrate flux (transmembrane velocity) on mass transport, an effect that is likely to be particularly important during SPTFF due to the requirement of high single-pass conversion. Trettin and Doshi [43] developed an approximate solution to the convection–diffusion equation that accounts for the effects of the transmembrane velocity under conditions where C_w_ >> C_b_, with the length-averaged flux given as:

(2) $$J_{v}=k_{o}{\left(\frac{C_{w}}{C_{b}}\right)}^{\frac{1}{3}}$$

where k_o_ is the overall mass transfer coefficient, and C_w_ and C_b_ are the nanoparticle concentrations at the membrane surface and in the bulk suspension, respectively. The mass transfer coefficient in the Pellicon^®^ 3 module was evaluated using a correlation presented by Da Costa et al. accounting for mixing generated by the spacer in the feed channel [44]:

(3) Sh=kodhD=0.664Re0.5Sc0.33dhlm0.5

where D is the nanoparticle diffusivity, calculated using the Stokes-Einstein equation with a particle radius of 61 nm as determined by DLS, giving $D=3.5\times 10^{-12}{\;m}^{2}/s$. Re is the Reynolds number (Re=Udhν), U is the feed channel velocity ($U=\frac{Q_{F}}{w*h}$), ν is the kinematic viscosity, Sc is the Schmidt number ($\mathrm{Sc}=\frac{\nu }{D}$), and $d_{h}$ = 100 µm and $l_{m}$ = 500 µm are the channel hydraulic diameter and spacer mesh length for the Pellicon^®^ 3 cassette with C-screen, as reported by Jabra et al. [45]. Equation (3) predicts a ½ power relationship between the permeate flux and feed flow rate (${\;J}_{v}\;\sim {\;Q}_{F}^{0.5}$), which is nearly identical to that observed for J_bl_ and only slightly below that for J_foul_ in Figure 4; this difference is likely due to the high conversion used in these experiments, which causes a significant variation in Q_F_ with position in the Pellicon^®^ 3 module.

The critical flux data can be used to estimate the value of C_w_ at which particle loss and fouling first become significant. For example, the data in Figure 3 give J_bl_ = 85 LMH and J_foul_ = 119 LMH at a feed flow rate of 30 mL/min (feed flux of 204 LMH), yielding C_w_ = 4.9 × 10^13^ and 1.1 × 10^14^ particles/mL, respectively. These correspond to particle volume fractions of 5 and 10%, calculated based on the volume of an individual spherical nanoparticle with a 122 nm diameter, both of which are almost 4 orders of magnitude greater than the nanoparticle concentration in the bulk suspension. Although the calculated value of the wall concentration for J_foul_ is somewhat lower than one might expect for the boundary layer to provide a significant resistance to flow, this discrepancy is likely a result of the approximate nature of Equation (2) in describing the appropriate concentration dependence at the very large concentration driving forces (C_w_/C_b_) seen in these experiments.

### 3.5. Conversion

**Figure 5:**
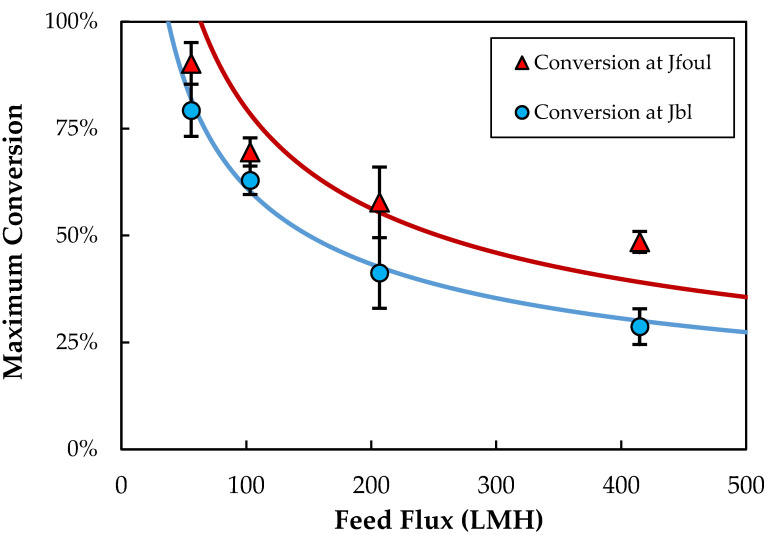
Maximum conversion corresponding to the boundary-layer (J_bl_) and fouling (J_foul_) critical fluxes as a function of feed flow rate for single Pellicon^®^ 3 module with a feed concentration of ≈2.5 × 10^10^ particles/mL. Error bars represent the range in flux values just above and below the transition point.

The effect of feed flow rate on the conversion, defined as the ratio of the permeate flow rate to the inlet feed flow rate, is examined in Figure 5. In each case, the conversion is evaluated at the critical flux associated with either boundary-layer accumulation (J_bl_) or fouling (J_foul_). The conversion decreases with increasing feed flow rate, since the critical flux varies with feed flow rate to a power-law exponent less than one. The solid curves in Figure 5 are the calculated values of the conversion based on the concentration polarization model (Equations (2) and (3)), using C_w_ = 4.5 × 10^13^ (4.3% by volume) and C_b_ = 2.7 × 10^10^ particles/mL for the boundary-layer critical flux and C_w_ = 8.7 × 10^13^ (8.3%) and C_b_ = 2.4 × 10^10^ particles/mL for the fouling critical flux. The bulk concentrations were taken as the average feed concentration prior to the critical flux across all experiments, and the wall concentrations were calculated using Equations (2) and (3). The model is in good qualitative agreement with the data, properly capturing the observed dependence of the conversion on the feed flow rate. The predicted conversion does exceed 100% at very low critical flux, since the model ignores the reduction in k_o_ (due to the reduction in retentate flow rate) and increase in C_b_ with length within the module.

**Figure 6:**
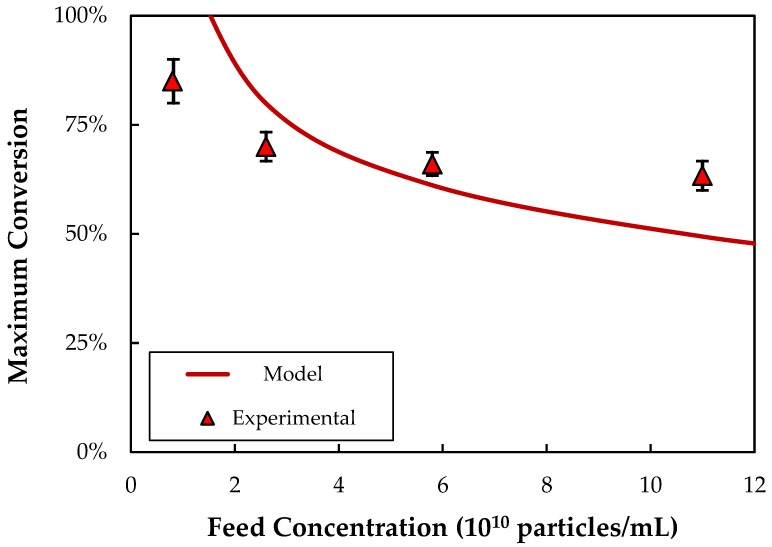
Predicted values of the maximum conversion (at J_foul_) compared to experimental data as a function of the nanoparticle feed concentration during SPTFF at a feed flux of 102 LMH.

The effect of the bulk nanoparticle concentration on the conversion corresponding to the fouling critical flux is examined in Figure 6, with the solid curve representing the model calculation. The experimental data were obtained by conducting flux-stepping experiments in single-pass mode, with the feed flow rate fixed at 15 mL/min (flux of 102 LMH) and the retentate and permeate exits routed to separate collection vessels. The filtrate flux was increased in a stepwise fashion by controlling the permeate exit pump. Following every flux step, any lost particles were recovered and recombined with the permeate and retentate to regenerate the feed suspension. The model is in qualitative agreement with the experimental data, although it does tend to predict a stronger dependence of J_foul_ on C_b_ compared to that observed experimentally. This likely reflects the approximate nature of the concentration driving force in the concentration polarization model (Equation (2)), as well as the assumption of constant nanoparticle diffusivity and suspension viscosity. The very high predicted conversions at nanoparticle concentrations below ~1.2 × 10^10^ particles/mL is again due to the large variation in Q_F_ and C_b_ within the module under these conditions, neither of which are accounted for in Equation (2), which simply uses the inlet values of Re and C_b_ and the length-average value of the filtrate flux.

### 3.6. Long-Duration SPTFF

**Figure 7:**
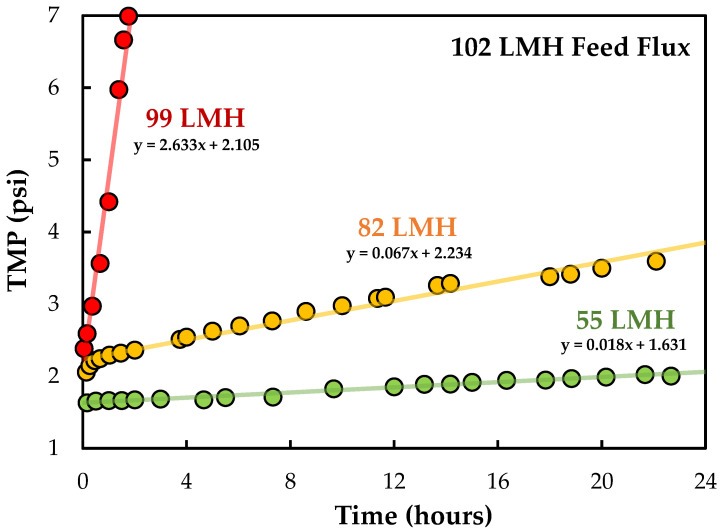
Long-duration SPTFF experiments performed in the Pellicon^®^ 3 cassette at a feed flux of 102 LMH and permeate fluxes of 55, 82, and 99 LMH. Equations represent the linear regression fits to the data.

Although SPTFF can be employed in batch operations, one of the major advantages of SPTFF technology is in the development of continuous bioprocesses that can be operated for long periods of time in a nearly steady state [16,46]. The effect of filtrate flux on the behavior of long-duration SPTFF experiments are shown in Figure 7. Data were obtained using a single Pellicon^®^ 3 cassette at a feed flux of 102 LMH and a nanoparticle concentration of ≈3 × 10^10^ particles/mL, which gives a critical flux of J_bl_ = 65 LMH and J_foul_ = 72 LMH based on the results in Figure 4. These conditions might be appropriate for the inline concentration prior to, or immediately after, other units in the downstream process where the increase in LV concentration or the reduction in process volume would enhance the overall productivity of the process. Experiments were performed in full recirculation mode with both the retentate and permeate exit streams recycled back to the feed reservoir (to conserve nanoparticles) at permeate fluxes of 55 and 82 LMH. At 99 LMH, the experiment was conducted in single-pass mode in order to keep the feed concentration fixed even as particles accumulated within the module.

The TMP for the run at 55 LMH increased by < 0.4 psi (from 1.6 to 2.0 psi) after 1360 min (~22 h) of continuous operation, and aside from an initial drop due to hold-up volume dilution effects, there was less than a 5% change in the nanoparticle concentration in the feed reservoir over the final 13 h of the experiment. The filtration at J_v_ = 82 LMH displayed a significantly larger increase in TMP (>1 psi), and there was a nearly 40% loss in particles; the feed reservoir needed to be periodically spiked with small volumes of a concentrated nanoparticle suspension to maintain the nanoparticle concentration at the target of 3 × 10^10^ particles/mL throughout the run. The nanoparticle loss was considerably higher for the run at 99 LMH, forcing this experiment to be conducted in single-pass mode, with 3 L of the nanoparticle feed suspension fed into the system over the 3 h filtration time. The TMP increased to more than 7 psi in this experiment, reflecting the high degree of fouling when the module is operated at J_v_ > J_foul_.

Although considerable care must be taken in extrapolating the results from these 24 h experiments to even longer operating times, the data in Figure 7 can be used to estimate the potential operating time for an SPTFF module assuming that there is a linear increase in TMP throughout the filtration. Results are summarized in Table 2 for the predicted operating time, without any membrane cleaning or regeneration, assuming that the SPTFF process is ended when the TMP = 20 psi; these times would be increased accordingly if the SPTFF operation were continued until a higher maximum TMP. The predicted operating time at J_v_ = 55 LMH, which is below the critical flux of J_foul_ = 72 LMH, was more than 6 weeks, but this was reduced to 11 days at J_v_ = 82 LMH and only 7 h at 99 LMH. However, it is important to note that these changes in operating flux also correspond to significant changes in the nanoparticle concentration factor achieved during SPTFF, with the concentration factor decreasing from 34 at 99 LMH to only 2.2 at 55 LMH. High conversion can only be achieved in the Pellicon^®^ 3 module (with J_v_ < J_foul_) by operating at a much lower feed flow rate or by using multiple Pellicon^®^ 3 modules in series to increase the overall amount of permeate removal.

The effect of module length on SPTFF performance was examined by using two Pellicon^®^ 3 modules in series, with Pellicon^®^ Single-Pass TFF diverter plates used to separate the feed and permeate channels. The retentate exit from the first channel was directly connected to the inlet feed of the second channel. Both permeate outlets were left open to the atmosphere, allowing independent measurements of the permeate fluxes from both cassettes. Pumps were placed on the inlet feed for the first cassette and on the retentate exit for the second cassette to control the overall flux. The two-cassette system provided stable operation at a feed flow rate of 30 mL/min corresponding to an overall feed flux of 102 LMH (calculated using the area of the two modules), with a total permeate flow rate of 21 mL/min (total flux of 72 LMH), giving a conversion of 71%. In this case, 88% of the permeate flow was obtained in the first cassette, with the remaining 12% obtained from the second cassette, giving an overall concentration factor of 3.3. The critical flux for the second cassette was estimated as J_foul_ = 53 LMH based on Equations (2) and (3), using the calculated inlet flow rate and the bulk nanoparticle concentration after accounting for the permeate removal in the first cassette. This is well above the measured permeate flux in the second cassette, which was 19 LMH (based on the area of only the second cassette). These results demonstrate that it is possible to design SPTFF processes to operate at higher conversion simply by increasing the total membrane length, e.g., by using two cassettes in series, with the filtrate fluxes reduced to minimize fouling and particle loss.

## 4. Conclusions

The data obtained in this study provide the first published investigation of the use of SPTFF for suspensions of ≈100 nm model nanoparticles with size and charge similar to that of commercially relevant lentivirus. Flux-stepping experiments identified the presence of two distinct values of the critical flux: one associated with particle accumulation within the module and one associated with membrane fouling. Both critical fluxes increased with increasing feed flow rate and decreasing nanoparticle concentration. This behavior was effectively described using a modified concentration polarization model, with the critical particle concentration at the membrane surface corresponding to a particle volume fraction of approximately 7.5%. Note that the degree of concentration polarization in this system, defined as the ratio of the wall to bulk nanoparticle concentrations, was ≈10^4^ due to the very dilute suspensions common in viral vector processing.

The model was used to identify conditions that should provide sustainable operation during long-duration SPTFF experiments. Data obtained during a continuous SPTFF experiment showed minimal increase in TMP or particle loss for operation below the boundary-layer and fouling critical flux values. Extrapolation of the TMP data suggests that it may be possible to operate under these conditions for upwards of 6 weeks, although this process only provided a concentration factor of 2.2. Higher concentration factors can be achieved by increasing the membrane path length, e.g., using a holder that allows operation with two cassettes in series.

The simple concentration polarization model developed in this study was in good agreement with experimental data at low to moderate conversions, but it was unable to describe the behavior at high conversions, since it does not account for the large variations in feed flow rate, bulk particle concentration, and local critical flux in the SPTFF module under these conditions. Future studies will be required to extend this framework to the design and operation of SPTFF systems that can provide high single-pass concentration factors, and to determine the generality of this approach for the concentration of actual lentivirus suspensions of interest in gene therapy manufacturing. Additionally, these studies will need to consider the effects of the shear flow, both in the module and the inlet/outlet ports, on the infectivity of the lentivirus.

## Figures and Tables

**Table 1:** Diameter and zeta potential of model nanoparticles and lentivirus. Nanoparticle size was determined in 10 mM PBS at pH 7.4. Zeta potential was evaluated in both 1 mM PBS at pH 7.4 and 50 mM tris, 5% sucrose, 50 mM L-arginine at pH 7.5.

	Nanoparticles	Lentivirus
Diameter (DLS)	122 ± 2 nm	123 ± 8 nm
Diameter (NTA)	104 ± 35 nm	113 ± 2 nm
Zeta potential (tris buffer)	−25.3 ± 1.1 mV	−17.7 ± 10.2 mV
Zeta potential (PBS)	−54.0 ± 1.3 mV	N/A

**Table 2:** Concentration factors and predicted operating times for SPTFF at a feed flux of 102 LMH based on a maximum pressure limit of 20 psi.

Permeate Flux	Concentration Factor	Predicted Operating Time
55 LMH	2.2	~6 weeks
82 LMH	5.1	~11 days
99 LMH	34.0	~7 hours

## Data Availability

The data presented in this study are available on request from the corresponding author.

## Supplementary Materials

The following supporting information can be downloaded at https://www.mdpi.com/article/10.3390/membranes13040433/s1: Figure S1. SEM image of carboxylate-modified polystyrene latex nanoparticles; Figure S2. Image of fouled membrane taken using a blue light transilluminator showing particle fluorescence on the membrane surface.
