# Supplementary material for: Single-Pass Tangential Flow Filtration (SPTFF) of Nanoparticles: Achieving Sustainable Operation with Dilute Colloidal Suspensions for Gene Therapy Applications
Source: Membranes (Basel). 2023 Apr 15;13(4):433. doi: 10.3390/membranes13040433 (PMC10143681; doi:10.3390/membranes13040433)
Supplement: Supplementary file 1 [file membranes-13-00433-s001.zip › membranes-2277939-supplementary.pdf]

membranes

MDPI

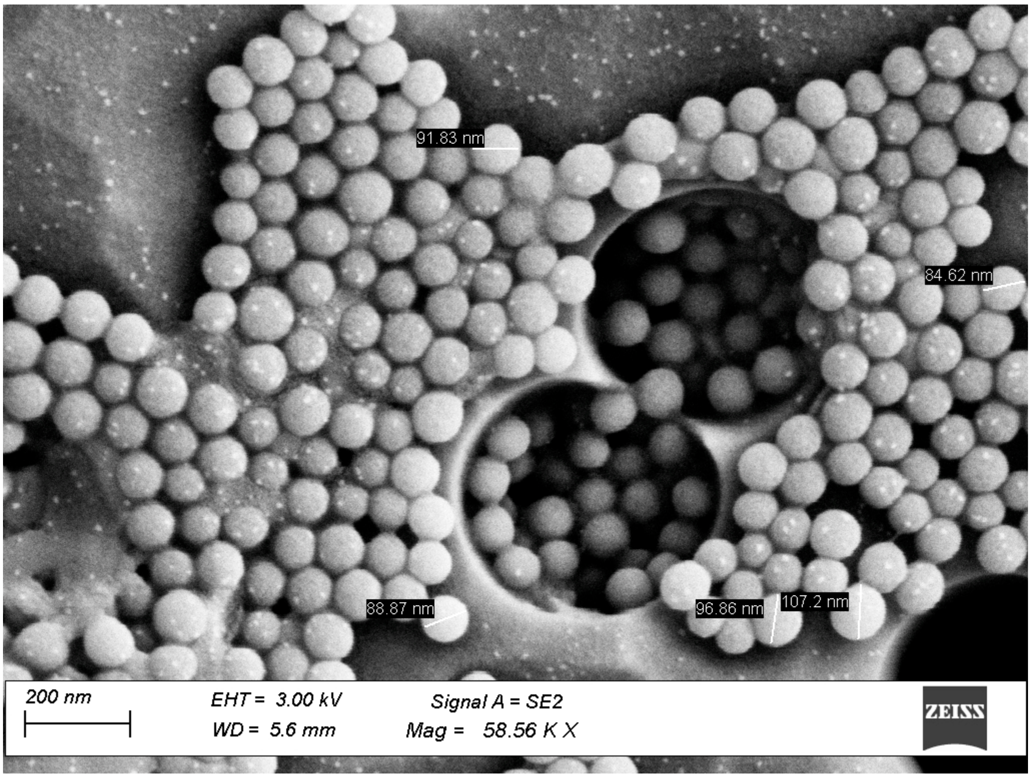

Figure S1. Image of carboxylate-modified polystyrene latex nanoparticles obtained via scanning electron microscopy (SEM). Particles were deposited on a 0.4 µm Isopore membrane (MilliporeSigma) and sputter-coated with gold/platinum using a Bal-tec SCD 050 sputter coater. The samples were then imaged using a Zeiss SIGMA VP-FESEM operated at 3.00 kV. The ~100 nm spheres in the image are the nanoparticles, while the larger holes are the ~0.4 µm membrane pores.

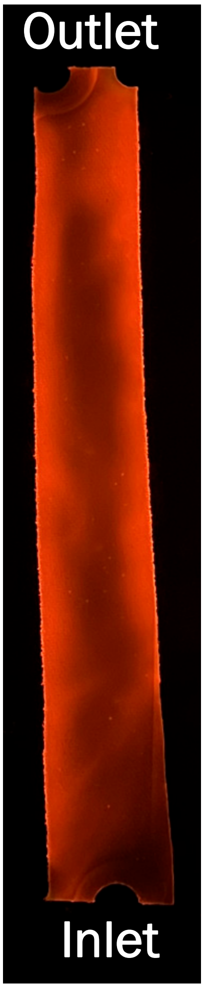

Figure S2. Image taken using a Dark Reader Transilluminator (Clare Chemical Research) of a 300 kDa Ultracel membrane removed from the Pellicon 3 cassette following experimentation. Fluorescence (in bright orange) observed on the membrane indicates nanoparticles deposited on the membrane surface during SPTFF.
